# Extracorporeal Membrane Oxygenation (ECMO): What We Need to Know

**DOI:** 10.7759/cureus.26735

**Published:** 2022-07-11

**Authors:** Hussein Rabah, Ali Rabah

**Affiliations:** 1 Internal Medicine, Northwell Health, New Hyde Park, USA; 2 Division of Electrophysiology, Beirut Cardiac Institute, Beirut, LBN

**Keywords:** veno-venous ecmo, critical care, respiratory support, cardiac support, veno-arterial ecmo

## Abstract

Extracorporeal membrane oxygenation (ECMO) is a form of circulatory support used in patients with refractory cardiac and/or respiratory failure. The main role of such support is to allow the lungs and heart to rest and heal while providing adequate oxygenation to vital organs. During extracorporeal support, the venous blood removed is decarboxylated, oxygenated, warmed, and infused back into the circulation. Physicians and nursing staff should be familiar with ECMO in order to provide the best care for critically ill patients. The aim of this paper is to review the technical aspects, indications, contraindications, complications, and management of both veno-venous (VV) and veno-arterial (VA) ECMO.

## Introduction and background

Extracorporeal membrane oxygenation (ECMO) is a form of circulatory support used in patients with cardiac failure, respiratory failure, or both, in whom conventional therapies have been exhausted. The venous blood removed is decarboxylated, oxygenated, warmed, and infused back into the venous (veno-venous (VV) ECMO) or arterial (veno-arterial (VA) ECMO) circulation. While the role of mechanical support during a respiratory failure is to allow the lungs to rest and recover while minimizing iatrogenic ventilator-induced lung injury (VILI), the goal of mechanical support in cardiac failure is to preserve end-organ perfusion while allowing myocardial rest and healing. Nowadays, there has been more interest in ECMO due to modernized equipment and experience. Consequently, its use has a 400% increase between the years 2006 and 2011 [1].

Despite the emergence of newer ventricular assist devices that are more suitable for long-term support, ECMO is simple to install, cost-effective, and widely available. However, it should only be performed by experienced clinicians familiar with its technical aspects, indications, and complications. This review describes the ECMO circuit and discusses its indications, contraindications, patient selection, and weaning.

## Review

Technical aspects

The fundamentals of ECMO have not changed during the past three decades. The circuit consists of a blood pump, oxygenator, and heat exchanger connected by drainage and return cannulas. The drainage cannula facilitates blood drainage from the patient into the pump, while the return cannula facilitates its return from the oxygenator into the patient. Drainage cannulation can be performed peripherally or centrally, whereas a return cannula is usually installed into a large central vein or the femoral artery, depending on the support indication. The resistance to blood flow depends on both the length and the diameter of the cannula. Shorter and broader cannulas decrease resistance, while longer and narrower ones increase resistance. The tubing system can be coated with biocompatible lining to limit the activation of the immune system and decrease the risk of inflammation, thrombosis, and bleeding [2].

During ECMO support, either roller or centrifugal blood pumps are the used mechanisms. Centrifugal pumps, although they carry an increased risk of hemolysis, have replaced roller pumps in most centers [3]. Centrifugal pumps convert rotational energy from a rotor spinning between 2,000 and 4,000 rounds/minute into kinetic energy, thus creating a pressure gradient driving the blood through the circuit. Once blood reaches the oxygenator, gas exchange is facilitated through a large polymer membrane that allows gas to diffuse across. Blood from the drainage side is pumped in on one side of the membrane, and gas is pumped into the other side. During this procedure, a water-based heater with the heat exchanger is incorporated into the oxygenator and rewarms the blood before being infused back into the patient’s circulation.

**Figure 1:**
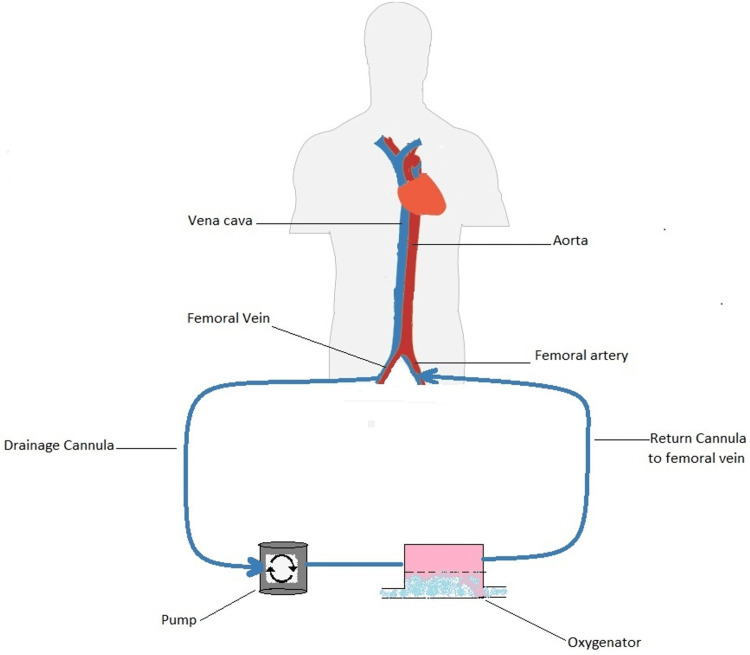
Veno-venous ECMO configuration. This figure shows how the venous blood is pumped into the ECMO circuit, oxygenated, and then infused back into the venous circulation.

The amount of oxygen provided via the artificial lung is a function of the blood flow, and its effects on systemic oxygenation depend on the type of cannulation and the function of the native lungs. In a veno-venous configuration (Figure 1), primarily used for respiratory failure, the improvement in arterial oxygenation is due to the increased oxygen saturation of the blood flowing through shunt areas of the lung. Significant improvements from oxygenation can therefore not be expected. In addition, as blood with high oxygen saturation reaches the pulmonary artery, the shunt fraction of the natural lung increases due to the loss of hypoxic vasoconstriction [4]. Despite this, the VV approach provides the organs with vital arterial oxygenation.

**Figure 2:**
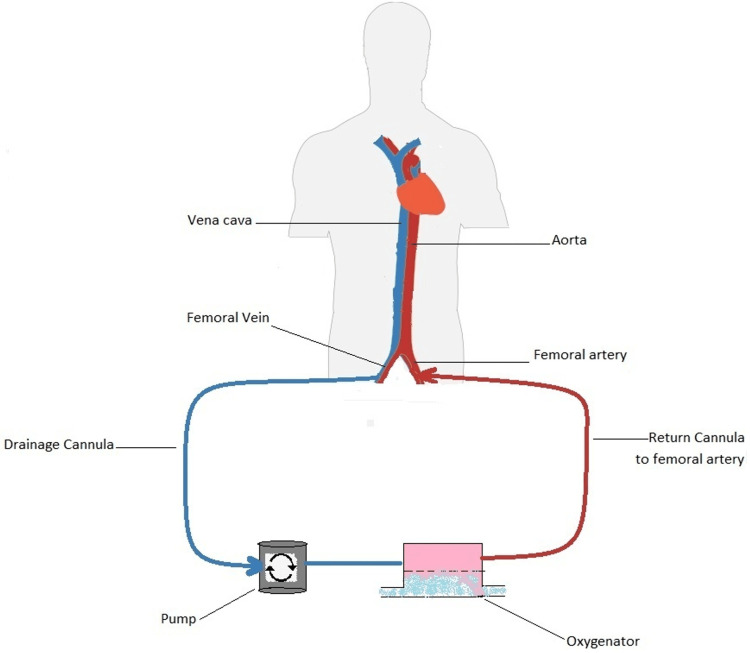
Veno-arterial ECMO configuration. This figure shows how the venous blood is pumped into the ECMO circuit, oxygenated, and then infused back into the arterial circulation.

The VA approach (Figure 2), mainly used for cardiac support, implies the drainage of venous blood, its oxygenation, and the subsequent input in the arterial circulation. There is no loss of hypoxic vasoconstriction in the pulmonary bed since no highly oxygenated blood enters the pulmonary artery, and therefore, there is no increase in the shunt fraction. In this modality, the oxygenated blood is mixed with the arterial blood to perfuse distal organs. However, this type of cannulation has significant disadvantages. First, it increases the risk of lower extremity ischemia as the femoral artery must be cannulated [5]. Moreover, there is a risk of differential hypoxia in which the brain and upper extremities are perfused by deoxygenated blood ejected from the native left ventricle, while the lower extremities are perfused by fully oxygenated blood provided by the ECMO [6].

The removal of CO2 is primarily a function of gas flow. The extracorporeal CO2 removal abolishes the need for ventilation and allows the lungs to rest [7]. Indeed, if the artificial lung’s primary purpose is protective ventilation, low extracorporeal blood flows are sufficient.

Indications for veno-venous ECMO

Veno-venous ECMO is an “artificial lung” that provides oxygenation and removes carbon dioxide. It is used in cases of severe respiratory failure and requires a functional native heart as it does not provide any cardiac support. The interest in this modality of respiratory support has increased since the H1N1 pandemic in the year 2009 [8,9].

Hypoxemic Respiratory Failure

In 2009, the Conventional ventilatory support versus Extracorporeal membrane oxygenation for Severe Acute Respiratory failure (CESAR) trial randomly assigned 180 patients with severe acute respiratory distress syndrome (ARDS) to an ECMO center or conventional ventilatory treatment. The authors defined severe ARDS as hypercapnic respiratory acidosis with an arterial pH < 7.20 or a Murray score greater than 3.0. It was concluded that referral to the ECMO center had significantly improved survival and recovery at six months compared to standard management (63% versus 47%) [10].

In 2018, the ECMO to rescue lung injury in severe ARDS (EOLIA) trial was conducted on 249 patients with severe ARDS. The patients received early veno-venous ECMO or conventional low-tidal volume low-pressure ventilation (which could introduce late ECMO as a rescue therapy). Severe ARDS was defined as partial arterial pressure of oxygen/fraction of inspired oxygen ratio (PaO2:FiO2) < 50 mmHg for >3 hours or PaO2:FiO2 < 80 mmHg for >6 hours. ECMO resulted in improved oxygenation, less chance of renal failure compared to other modalities of treatment (46% versus 21%), and fewer cases of ischemic stroke (0% versus 5%). The final analysis concluded that the 11% difference in 60-day mortality, while favoring early ECMO, was not significant (35% versus 46%) [11].

The Berlin consensus report on acute respiratory distress syndrome (ARDS) suggests ECMO in severe respiratory failure (PaO2:FiO2 < 70) despite optimization of ventilatory settings [12]. In addition, a meta-analysis reported that patients receiving veno-venous ECMO had lower 60-day mortality (34% versus 47%) [13].

Hypercapnic Respiratory Failure

ECMO can facilitate the removal of carbon dioxide and reduce ventilator-induced lung injury caused by barotrauma. The SUPERNOVA trial assessed the usefulness of extracorporeal carbon dioxide removal as a component of ultra-protective ventilation (tidal volume 3-4 mL/kg and plateau pressure at or above 25 cm H2O). The primary outcome was the number of patients who successfully achieved a tidal volume of 4 mL/kg of predicted body weight with arterial carbon dioxide not increasing more than 20% from baseline, in addition to an arterial pH > 7.30. It was evident that protective ventilation supported by extracorporeal carbon dioxide removal is feasible in moderate ARDS. However, there has been a relatively high incidence of circuit clotting, hemolysis, and bleeding [14].

Bridge to Lung Transplant

Other studies showed that ECMO could serve as a bridge for lung transplantation. In a study of 40 patients with interstitial lung disease and respiratory failure, 21 were treated with ECMO. Eventually, six patients treated with support were bridged to lung transplant [15].

Table 1 summarizes the indications of veno-venous ECMO.

**Table 1:** Summary of veno-venous ECMO indications.

Indications for veno-venous ECMO
Hypoxemic respiratory failure and one of the following:
Murray score greater than 3
PaO2:FiO2 < 50 mmHg for more than three hours
PaO2:FiO2 < 80 mmHg for more than six hours
PaO2:FiO2 < 70 mmHg despite optimization of ventilator settings
Hypercapnic respiratory failure with pH < 7.2 despite optimization of ventilator settings
Bridge to lung transplant

Indications for veno-arterial ECMO

When applied in the veno-arterial configuration, the ECMO bypasses the pulmonary circulation while providing cardiac support. This allows for organ perfusion while the heart rests and recovers. Such an approach is a bridge to “recovery, decision, or transplant” (Table 2).

Cardiopulmonary Arrest

Not uncommonly, the VA-ECMO is used during resuscitation from cardiac arrest. Extracorporeal cardiac pulmonary resuscitation (ECPR) utilizes VA-ECMO in patients who are unable to achieve a sustained return of spontaneous circulation, which is defined as 20 consecutive minutes without a need for chest compressions [16]. It is suggested that ECPR application in selected patients improves both short- and long-term survival compared to conventional CPR [17]. In order to attain a favorable neurological outcome, the initiation of extracorporeal cardiac support should be within the first 21 minutes of the cardiac arrest [18]; otherwise, the prognosis is poor, especially when deployed after 30 minutes [19]. ECPR should not be used in patients with unwitnessed cardiac arrest, resuscitation effort of more than 60 minutes, or anoxic brain injury.

Cardiogenic Shock

Extracorporeal support has also been employed in the context of cardiogenic shock (CS), which is clinically defined as systemic hypoperfusion caused by acute or acute on top of chronic heart failure. Hemodynamically speaking, it is described as a systolic blood pressure < 90 mmHg (or the need for supportive measures to maintain it above 90 mmHg), a cardiac index below 2.2 L/minute/m^2^ of the body surface area, and a pulmonary-capillary wedge pressure of 15 mmHg and above [20]. The etiology leading to CS could be acute myocardial infarction, acute decompensated heart failure, chronic decompensated heart failure, pulmonary embolism with right ventricle failure, myocarditis, post-heart transplant graft rejection, or post-cardiotomy syndrome. With pharmacological treatment alone, recovery is rare, and the mortality has been as high as 51% [21]; thus, VA-ECMO should be offered to patients with reversible causes of CS. Also, ECMO has been used in circulatory support in high-risk coronary interventions [22,23] and is becoming a rescue means for acute hemodynamic compromise resulting from a variety of invasive procedures, such as transcatheter aortic valve replacement [24].

Ventricular Tachycardia

Moreover, extracorporeal life support (ECLS) has been applied successfully to patients with refractory ventricular tachycardia (VT). In a study conducted from January 2002 to December 2004, a total of 11 patients were enrolled, from which nine patients received veno-arterial mode ECLS support in an attempt to terminate refractory ventricular arrhythmias. Ventricular tachycardias terminated rapidly following ECLS deployment in all patients, and nine were discharged with no evident recurrent VT or the need for internal cardiac defibrillator implantation [25].

Table 2 summarizes the indications of veno-arterial ECMO.

**Table 2:** Summary of veno-arterial ECMO indications.

Indications for veno-arterial ECMO
Resuscitation during cardiopulmonary arrest
Cardiogenic shock of the following etiologies:
Acute coronary syndrome
Acute heart failure
Decompensated heart failure
Fulminant myocarditis
Pulmonary embolism with right heart failure
Post-heart transplant graft rejection
Post-cardiotomy
High-risk coronary interventions
Hemodynamic instability resulting from TAVR
Refractory ventricular tachycardia
Bridge to cardiac transplant or ventricular assist devices

Contraindications for ECMO

There are no absolute contraindications for ECMO, but its use should be based on a case-by-case decision [26]. However, there are situations in which the benefit of the ECMO is questionable. Circulatory support should not be used in cases of unwitnessed cardiac arrest, resuscitation effort of more than 60 minutes, severe irreversible noncardiac organ damage limiting survival (anoxic brain injury or end-stage malignancy), or when heart failure is irreversible and ventricular assist devices or transplantation are not feasible. Hemodynamic compromise caused by aortic dissection is also among such contraindications in which cannulation should not be attempted.

Relative contraindications include uncontrollable bleeding, severe coagulopathy, or contraindication to anticoagulation, in addition to limited vascular access such as obesity and severe peripheral vascular disease (Table 3).

**Table 3:** Summary of ECMO support contraindications.

Contraindications for ECMO support
Absolute contraindications
Unwitnessed cardiac arrest
Resuscitation effort of more than 60 minutes
Irreversible noncardiac organ damage limiting survival (anoxic brain injury or metastatic cancer)
Irreversible heart failure not amendable to transplantation or ventricular assist devices
Aortic dissection
Irreversible lung disease not amendable to transplantation
Uncontrollable bleeding
Severe coagulopathy
Contraindication to anticoagulation

Complications

Complications during ECMO treatment are frequent. The main complications reported are failure of the oxygenation membrane, intracranial hemorrhage (ICH), acute kidney injury (AKI), infections, limb ischemia, differential hypoxia, and ECMO lung.

Oxygenation Membrane Failure

It is reported that the incidence of failure of the oxygenation membrane in adult patients is 5.8% in VV-ECMO and 2.8% in VA-ECMO [27]. The primary reason behind this failure is clot formation within the oxygenator. A drop in the partial pressure of post-oxygenator oxygen, an increase in transmembrane pressure gradients, a progressive increase in the fresh gas flow, and sudden increases in D-dimer levels should point toward oxygen membrane failure [28]. Inadequate anticoagulation, disseminated intravascular coagulation, and heparin-induced thrombocytopenia are the main factors contributing to circuit thrombosis and should be identified and managed appropriately.

Intracranial Hemorrhage

According to the Extracorporeal Life Support Organization (ELSO) report, the incidence of intracranial hemorrhage (ICH) and ischemic stroke in adult patients on VV-ECMO is 2.1% and 3.1%, respectively, while it is 1.6% and 3.1%, respectively, in patients on VA-ECMO [27]. Alteration in hemostasis is proposed as a principal mechanism behind ECMO-induced ICH. ECMO support itself results in thrombocytopenia (54%), factor XIII deficiency (88%), acquired von Willebrand syndrome (79%), and fibrinogen deficiency (40%) [29]. This deficiency in clotting factors is due to the shear stress induced by continuous flow across the device and the contact between the patient’s blood with the ECMO circuit that leads to the activation of the coagulation cascade. In addition, inadequate control of anticoagulation, acute kidney injury, the need for renal replacement therapy (RRT), and the female gender are associated with an increased risk of ICH [30].

Acute Kidney Injury

The incidence of acute kidney injury(AKI) in adults managed with ECMO is 78% in patients with respiratory failure [31] and 81% in post-cardiotomy patients [32]. Adults with AKI have a fourfold increase in mortality compared with non-AKI patients [31].

The pathophysiology of such an insult is not well understood. Rapid hemodynamic fluctuations caused by the ECMO and pressors that alter renal blood flow leading to ischemia-reperfusion-associated AKI is a proposed hypothesis [33], in addition to systemic inflammation, hypercoagulable state, and hemolysis associated with ECMO support.

Infections

Patients supported with ECMO usually have a complicated and prolonged hospitalization. ECMO itself, as a foreign body, increases the risk of nosocomial infections. In addition, such patients frequently require invasive hemodynamic monitoring and interventions. Indeed, they become susceptible to septic complications, commonly bloodstream and surgical site infections.

Analysis of the Extracorporeal Life Support Organization (ELSO) registry concluded that older age, longer duration of ECMO support, and longer post-ECMO ventilation increase the risk of infections. Only 6.1% of patients requiring bypass for ≤7 days had an infection, whereas 30.3% of those requiring ECMO for >14 days were diagnosed with an infection. The most common causative organisms were coagulase-negative staphylococci, *Candida*, and *Pseudomonas* [34]. Treatment should be directed against the causative organism, and the prophylactic use of antibiotics is not recommended [28].

Limb Ischemia

Cannulation of the femoral artery while securing the VA-ECMO access carries a risk of lower extremity ischemia [5]. The risks are more significant with larger cannulas (>20 Fr), females, young patients, and the presence of peripheral arterial disease. Placement of antegrade distal perfusion catheter in the proximal superficial femoral artery at the time of ECMO cannulation of the common femoral artery has been shown to reduce the incidence of limb ischemia [35,36].

Other complications include dissection, pseudoaneurysm, and retroperitoneal bleeding. Complications can also occur during the ECMO support or while performing ECMO separation.

Differential Hypoxia

During VA-ECMO support maintenance via the femoral artery, there is a risk of differential hypoxia in which the brain and upper extremities are perfused by deoxygenated blood ejected from the native left ventricle while the lower extremities are perfused by fully oxygenated blood provided by the ECMO [6]. This disastrous complication can lead to anoxic brain injury if not recognized and managed appropriately. Clinically, the patient’s head and upper extremities are cyanotic, while the lower extremities are pink and well perfused. This syndrome is diagnosed by low partial oxygen pressure measured in the right upper extremity and is managed by cannulation of the internal jugular vein and infusing oxygenated blood into the right atrium. Such a circuit is called veno-venoarterial ECMO [6].

ECMO Lung

The retrograde ejection of blood through the femoral artery and aorta during VA-ECMO support increases the left ventricular afterload. In patients with cardiogenic shock, this worsens the myocardial contractility, increases the filling pressures, and decreases cardiac output. Eventually, pulmonary congestion will develop or worsen, leading to a more systemic inflammatory response that promotes more acute lung injury and even alveolar hemorrhage.

The ECMO lung can be treated using inotropes to overcome the increased afterload by improving cardiac contractility. Intra-aortic balloon pump (IABP) or Impella can be deployed to unload the left ventricle. If all measures fail, percutaneous atrial septostomy or surgical decompression cannula can be attempted [37].

Adjustment of ECMO settings

Patients on ECMO support should be medically managed similar to patients not on any circulatory assistance. This includes inotropes, vasopressors, vasodilators, crystalloids, and blood product transfusions.

The initial blood flow should be maximized and then lowered to maintain an oxygen saturation of 80% and above. While applying VA support, hemodynamics are a function of blood flow and vascular resistance. Thus, organ perfusion is achieved by determining the adequate circuit blood flow needed and optimizing the cardiac function in this setting.

Systemic perfusion is guided based on venous oxygen saturation (SvO2) measures with a target of 70%. Supposing the mixed venous oxygen level is below the target, blood flow should be enhanced by increasing the VA-ECMO circuit blood flow, improving the native cardiac output using inotropes/pressors or unloading methods (IABP). If more intravascular volume is needed to achieve adequate flow, infusing crystalloids and blood products should be considered. In contrast, there is no cardiac support provided by the VV-ECMO circuit; therefore, there is no need to assess the perfusion using SvO2 in this setting.

As for the partial pressure of carbon dioxide (PaCO2), it is controlled by the sweep gas flow. CO2 diffuses 20-fold faster than O2; thus, large amounts of CO2 can be exchanged through the membrane lung even when the flow through the circuit is low. When the “sweep gas” flow rate is the same as the blood flow rate, the amount of oxygen added and the CO2 removed are approximately equal, but when the ratio of gas flow to blood flow is increased, there is no increase in oxygenation, but CO2 clearance increases in proportion to the gas flow. Thus, when blood decarboxylation is desired, the higher the gas/blood ratio, the more CO2 is removed. The gas flow is then adjusted to maintain a pH close to 7.40 and a partial pressure of carbon dioxide (PaCO2) of 40 mmHg. Hence, in patients with refractory hypercapnic respiratory failure, low extracorporeal blood flow is sufficient [38].

Ventilation during ECMO support

When VV configuration is used, some of the venous blood is pumped into the circuit. As the blood passes through the oxygenator, it becomes fully saturated. This portion of the blood is then infused back into the native venous circulation and becomes mixed with desaturated blood in the right atrium, pumped into the lungs, left ventricle, and then native arterial circulation. The goal is arterial oxygen saturation of 80%. This will maintain tissue oxygenation even with mild-to-moderate hypoxemia if the cardiac output is adequate and there is enough hemoglobin to deliver the oxygen. Therefore, there is no need for pulmonary gas exchange, and the ventilator setting should be adjusted to allow lung recovery and prevent iatrogenic ventilator-induced lung injury (VILI). For this purpose, the ELSO guidelines recommend ventilator “rest settings” for patients on VV-ECMO support: pressure-controlled ventilation, inspiratory pressure of 25 cm H2O, positive end-expiratory pressure of 15 cm H2O, inspiratory to expiratory (I:E) ratio of 2:1, below 40% FiO2, and a respiratory rate of five breaths/minute. The usual tidal volume is 4 mL/kg of predicted body weight. However, a tidal volume of less than 1.5 mL/kg of predicted body weight has been used [39]. The patient should be moderately to heavily sedated during the first 24 hours. After one day of ECMO support, it is recommended that sedation be minimal, given that the patient is stable and improving hemodynamically [38]. The ICU staff should be educated regarding the permissive hypoxemia in patients on VV-ECMO support, and the temptation to adjust ventilator settings to improve oxygenation should be resisted.

In contrast, during the VA-ECMO support, the oxygenation of the coronary arteries, aortic root, upper extremities, and brain is more dependent on the patient’s native cardiopulmonary function, while the perfusion of the organs distal to the mid aorta is maintained mainly by the ECMO circuit. Therefore, using “rest ventilation” as in VV support will put the patient at risk of “differential hypoxia syndrome“ causing myocardial hypoxia and anoxic brain injury. Ventilator settings should be adjusted to achieve adequate pulmonary gas exchange, and a higher PEEP and FiO2 may be necessary. In central subclavian artery cannulation, the risk of this complication is eliminated.

Weaning from ECMO

In patients on VV support, improvements in radiographic appearance, pulmonary compliance, and arterial oxyhemoglobin saturation indicate that the patient might maintain satisfactory gas exchange and is ready to be weaned from ECMO. The blood flow should be decreased gradually and slowly over hours until 2 L/minute while assessing the patient. Once the blood flow is 2 L/minute, the sweep rate of the gas across the oxygenator should be decreased slowly to zero. This will eliminate the ECMO contribution to gas exchange. During the weaning trial, the optimal ventilator settings (PEEP, RR, and FiO2) should be determined to facilitate pulmonary gas exchange. If the patient remains hemodynamically stable and the arterial partial pressure of carbon dioxide is below 50 mmHg, the weaning is completed, and decannulation can be attempted [38].

For patients on VA-ECMO, the decision to proceed with weaning depends on the clinical and hemodynamic evidence of improvement and recovery. Recovery of the cardiac function can be assessed by echocardiography, decrease in the need for inotropes/pressors, mean arterial pressure of more than 60 mmHg, and stable heart rate and rhythm. The combination of improved radiographic parameters, lung compliance, and (PaO2)/fraction of inspired oxygen (FiO2) > 200 mmHg on 30%-40% FiO2 predicts pulmonary improvement. If those criteria are fulfilled, a weaning trial can be initiated by decreasing the ECMO support by reducing the flow over hours to 2 L/minute. If vital signs and hemodynamic parameters remain stable, flow can be decreased to 1 L/minute. After one hour, the parameters should be reassessed. The patient can be removed from ECMO support if the left ventricular ejection fraction is >20% with adequate right ventricular function, central venous blood oxygen content (CVO2) is >60%, pH is >7.30 on a sweep rate of 1-2 L/minute, and PaO2:FiO2 is >200 with FiO2 of <60% [40]. If the patient cannot be weaned off the ECMO, cardiac transplant or ventricular assist devices should be considered.

## Conclusions

Extracorporeal membrane oxygenation is currently being increasingly used as a support device in critically ill patients with refractory cardiac and/or respiratory failure. It is simple to install, cost-effective, and widely available. However, it should only be performed by experienced clinicians familiar with its technical aspects, indications, and complications. The main goal of ECMO support is to allow the heart/lungs to recover while maintaining oxygenation/perfusion to vital organs. Patient selection is important in order to determine the ECMO configuration, and it is crucial that intensive care unit staff be familiar with this type of circulatory support since its management is a collaboration between physicians, technicians, perfusionists, and nurses.
